# Suicide Attempts Among French and Brazilian Adolescents Admitted to an Emergency Room. A Comparative Study of Risk and Protective Factors

**DOI:** 10.3389/fpsyt.2020.00742

**Published:** 2020-08-06

**Authors:** Natalia C. Rufino, Bojan Mirkovic, Angèle Consoli, Hugues Pellerin, Juliana P. M. Santos, Thiago M. Fidalgo, Priscille Gerardin, Dartiu X. Silveira, David Cohen

**Affiliations:** ^1^ Department of Psychiatry, Universidade Federal de São Paulo, São Paulo, Brazil; ^2^ Service de Psychiatrie de l’Enfant et de l’Adolescent, CHU Charles Nicolle/CH Le Rouvray, Normandie Université, Rouen, France; ^3^ Département de Psychiatrie de l’Enfant et de l’Adolescent, AP-HP, Groupe Hospitalier Pitié-Salpêtrière, Paris, France; ^4^ GRC-15, Approche dimensionnelle des épisodes psychotiques de l’enfant et de l’adolescent, Faculté de Médecine, UPMC, Sorbonne Université, Paris, France; ^5^ CNRS UMR 7222 “Institut des Systèmes Intelligents et Robotiques”, Sorbonne Université, Paris, France

**Keywords:** adolescent, suicide attempt, coping, spirituality, attachment style

## Abstract

**Background:**

Suicide is the second most common cause of preventable mortality among Brazilian and French adolescents. The aim of the current study was to compare the main risk and protective factors associated with a suicide attempt (SA) and to highlight differences based on geographical characteristics.

**Method:**

We compared a Brazilian sample (N = 45) of adolescents admitted to the emergency room of a public hospital in São Paulo for SA to a French sample (N = 320) of adolescents hospitalized for SA across 5 paediatric departments. Then, we ran several multivariate models to examine how each selected variable was related to geographic origin and to the other selected variables linked to geographic origin.

**Results:**

The two samples presented no significant differences regarding gender, age or schooling. Both samples had high rates of depressive disorders, anxiety disorders, substance use, disruptive disorders, borderline psychopathology, and lifetime SAs. However, the Brazilian sample presented significantly higher levels of psychopathology and had more insecure attachment relationships (fearful and detached), whereas the French sample had a more secure attachment style. Brazilian adolescents had more recourse to spiritual beliefs and spiritual support, whereas the French adolescents had higher scores on the Reasons for Living Inventory and used more help-seeking strategies from their social network, mainly close friends. Multivariate models showed that two productive coping strategies (seeking spiritual support and social action) and the dependence score were significantly associated with membership in the Brazilian cohort, whereas a secure attachment style and depression severity (evaluated by the Beck Depression Inventory) were significantly associated with membership in the French cohort.

**Conclusion:**

Despite presenting similar psychopathologies, Brazilian adolescents presented a more insecure attachment style and used the religious kind of coping more commonly than their French counterparts. We hypothesize that religion may compensate for the social vulnerabilities present in a middle-income country such as Brazil. More transcultural studies may help to elucidate this phenomenon.

## Introduction

Suicide is the second leading cause of death in youth (10 to 24 years) worldwide (1) and a total of 78% of suicides occur in low- and middle-income countries (2). A recent meta-analysis of 686,672 children and adolescents found that the aggregate lifetime and 12-month prevalences of suicide attempts were 6% and 4.5%, respectively (3). The aggregate lifetime and 12-month prevalences of suicidal plans were 9.9% and 7.5%, respectively. The aggregate lifetime and 12-month prevalences of suicidal ideation were 18% and 14.2%, respectively.

In Brazil, suicide is the fourth leading cause of death in young people between the ages of 15 and 29 years old (4). Epidemiological data generated from the Mortality Information System (SIM) of the Brazilian Ministry of Health in 2017 found a suicide rate of 1.1/100.000 among children and adolescents (between the ages of 1 and 19 years old). The prevalence of suicide attempts (SA) in adolescents ranges from 5.9% to 10% (5, 6). Even if Brazil has relatively low suicide rates among young people (ranked 93^rd^ out of 195 countries and territories) (2), there was a 13% increase in the nationwide adolescent (10 to 19 years old) suicide rate from 2006 to 2015 and a 27% increase in the São Paulo metropolitan area (6). In France, suicide is the second leading cause of death among 15- to 24-year-olds. According to the latest national data available, the suicide rate for adolescents aged 15-19 years old is 4.1 per 100,000 inhabitants (7). In 2016, European data reported an average suicide rate of 4.29 per 100,000 inhabitants for subjects aged 15 to 19 years old among the 28 EU countries, with a significantly higher rate among boys than among girls (2.1 vs. 5.9 per 100,000) (8).

Suicidal behavior is considered to result from multiple biopsychosocial interactions (9). On the one hand, an individual’s neurobiological (10), genetic (11), cognitive and emotional (12, 13) characteristics may affect suicidal behavior; on the other hand, an individual’s environment in the broad sense of the term, namely, situations regarding adversity (14), family and social relationships (15, 16) life time trajectory, culture, or spirituality (17), may affect suicidal behavior. In addition to the “opposition” of biological vs. environmental factors, several authors have proposed the modelling suicide behavior risk by the “combination” of time-related risk and protective factors (18). Protective factors are attributes of people, environments, situations and events that appear to moderate psychopathological predictions based on individual risk status (19, 20). Recent studies have highlighted the role of protective factors in the assessment of suicide risk, namely, productive coping skills, reasons for living, social support, or spirituality (18, 21–23). Other protective factors include religion (24, 25) and the resolution of participants (26).

In an integrative model, the evaluation of risk factors remains crucial because a factor is only protective insofar as it moderates the impact of the risk factor(s). For a given individual, protection and risk factors interact in an inversely proportional manner. Thus, when faced with a risk situation, the individual will produce a more positive response if the “protection” pole is dominant and a more negative response if the “vulnerability” pole is dominant (18). In addition, the role of cultural references for interpreting protective factors is matter of debate and has not been studied in detail, as it requires large studies in different cultural settings. To date, only geographical variations have been described at a macro level (e.g., more suicides in rural areas (vs. urban areas) of China (27); a higher proportion of suicides in America and Europe (vs. Asia) meeting criteria for psychiatric disorders (1). Cultural and socioeconomic aspects related to geographical location also play an interchangeable role, as they influence both risk and protective factors. Being part of an ethnic minority is described as a risk factor (28–30), and socioeconomic status could be a protective factor—as it is related to a higher educational level and greater social support (18, 18)—or a risk factor (30, 31) for adolescents who exhibit SB. It is also important to consider that socioeconomic barriers and cultural characteristics could have a considerable impact on adolescents’ treatment, especially those with suicidal risk behavior (32). There are differences in risk factors for suicide between adults and adolescents. Adolescents who attempted suicide have been shown to have comparatively more relationship problems but fewer medical problems than adults (33).

Studies of the prevalence of child and adolescent psychiatric disorders in Brazil have reported similar levels of psychopathology as other international studies but slightly higher levels than findings from developed countries, which corroborates previous epidemiological findings suggesting the universality of psychiatric disorders in children and adolescents across cultures (34, 35). Risk and protective factors associated with SB have been well established in studies from developed countries, but these factors remain underexplored in low- and middle-income countries. Although adolescent suicidal behaviors vary across countries, there is a consistent set of risk factors for suicidal behaviors across all regions and most countries, and little is understood about protective factors and their moderating effects on suicide risk in adolescence (36). In 2002, an intervention study on suicidal behavior—The Suicide Prevention—Multisite Intervention Study on Suicidal Behaviors (SUPRE-MISS)—was initiated by the World Health Organization. The survey was conducted in seven culturally diverse low- and middle-income countries around the world, including one site in Brazil, and provided valuable information regarding socioeconomical and cultural characteristics on SB among adults (37–39), including the role of subjective religiosity (25) and the impact of brief interventions in the emergency department (39) as a protective factor in the Brazilian sample. A process of seeking relief in new religious affiliations that is probably occurring in Brazilian society among subgroups with previous minor psychiatric symptoms has already been described by Dalgalarrondo et al. (40).

There is still much to explore regarding protective factors that are related to cultural and socioeconomic aspects, such as religiosity, coping styles, reasons for living, attachment and stressful life events, especially in the Brazilian adolescent population.

In the current article, we propose a comparison between a Brazilian clinical population of adolescents with suicidal behaviors with a French clinical sample. We aim to identify more well-established associated risk and protective factors by using the same standardized tools to evaluate these samples.

We hypothesized that factors related to the psychopathology of adolescent suicide behaviors (mood disorders, conduct disorders, borderline psychopathology, substance abuse, impulsivity, hopelessness, and lifetime suicide attempt) would be common to both populations, whereas some protective factors (reason for living, spirituality, coping skills or attachment style) would be different based on geographical origin.

## Method

### Participants

The study was conducted in two countries. The inclusion criteria were similar in both samples and were based on WHO definitions and the most recent classifications of suicidal behaviors. We considered the main inclusion criterion to be showing “a suicide attempt” only if the intent to die was manifested (41). Subjects committing nonsuicidal self-injury without the intent to die were excluded. Other inclusion criteria included being an adolescent aged 12-18 years, being able to understand the purpose of the study, and the absence of prominent mental retardation or organic brain damage. Participants were informed that their answers would remain confidential, and written informed consent for study participation was obtained from participants as well as both parents or guardians.

The French sample included 320 adolescents aged 12 to 18 who were admitted to 1 of 4 paediatric departments following a SA between January 2011 and December 2014 -detailed protocol in previous paper (42). For the purpose of this transcultural study, we recruited a Brazilian sample using identical inclusion and exclusion criteria and a similar assessment protocol. Brazil is a large country that is characterized by high social disparities between regions. São Paulo, the country’s financial centre, is the sixth largest metropolitan area in the world, with a population of over 12 million habitants.

In Brazil, unlike in France, access to mental health care appear to be hampered by multiple socio-cultural and economic factors (43). The Unified Health System (Sistema Único de Saúde), conceived in the late 1980s has enabled substantial progress towards Universal Health Coverage in Brazil. However, structural weakness, economic and political crises and austerity policies have limited its growth and sustainability and outcomes (44).

Therefore, in Brazil, most adolescents are not hospitalized after a SA. Second, few public facilities are associated with a research team. Consequently, for the São Paulo centre, we chose a public centre associated with a research team to avoid socioeconomic biases and to avoid patients who lacked access to free care. From April 2016 to November 2017, 61 adolescents were recruited to participate in this study. Eleven refused to participate, and 4 withdrew their consent during the evaluation process. In total, 45 adolescents aged 11 to 18 were admitted to the emergency department of a university hospital in São Paulo, Brazil, after an SA and were included in the study.

### Procedures

For the French sample, assessments were performed during a 1-week inpatient stay. The procedure has been detailed in Mirkovic et al. (42). For the Brazilian sample, access to care is different from that in France, and the inpatient setting for adolescent psychiatric care is more limited. Consequently, Brazilian adolescents could not be assessed in the same way. On admission to the emergency department, participation in the study was systematically proposed to all adolescents who met the inclusion criteria by the psychiatrist in charge. After obtaining the consent of the adolescent and their parents, the research team contacted the patient and his/her family. The evaluation was conducted within 1 month of the visit to the emergency department by psychiatrists trained in conducting the evaluation. To avoid tiring the patients, the evaluation was carried out over multiple interview sessions. The investigators were psychiatrists with more than 3 years of clinical experience. All the evaluation reports were analyzed individually by our study group, and final diagnoses were made after consensus among the group.

### Measures

In addition to sociodemographic data, we explored axis I psychiatric disorders using the Scale of Mood Disorders and Schizophrenia for Children and Adolescents of School Age, Current and Past Episodes version (Kiddie-SADS-PL) (45). The interview protocol was translated into French by Mouren-Siméoni et al. (46) and into Portuguese by Brasil HHA et al. (47). The Columbia Suicide Severity Rating Scale (C-SSRS) (41) was used to quantify the severity of suicidal ideation and behavior. This scale allows for the assessment of suicidal behavior and suicidal intentionality. It was completed by the clinician based on the clinical interviews conducted with the adolescents. The Beck Depression Inventory, Second Edition (48) was used to evaluate the severity of depression symptoms. It comprises 21 items rated on a 4-point scale. Scores range from 0 to 63. The Beck Hopelessness Scale (49), a self-report scale designed to measure levels of hopelessness, is composed of 20 true-false items. The Eysenck Questionnaire (50) was used to rate impulsivity. This 24-item self-administered questionnaire was validated for young people aged 8 to 17.

The Ab-Diagnostic Interview for Borderline Patients (Ab-DIB) is a DIB-R–derived self-report to assess impulsiveness among adolescents as well as the effect and cognitive components of the borderline construct (51, 52). Substance use and misuse were assessed with the Dependence Questionnaire for Adolescents (DEP-ADO) (53).

We also explored several variables we hypothesized as potential moderators of suicide risk or relapse (18). 1) We used the Adolescent Coping Scale (ACS), which was designed and validated for adolescents aged 12 to 18. The ACS comprises 77 items grouped into 18 subscales representing 18 specific coping strategies (54). 2) We used the Spirituality Scale, which assessed the broad construct of spirituality and was developed by Delaney et al. (55). This scale goes beyond religious practices and assessed 3 key relational aspects: connection with self (personal), with others (interpersonal), and with the divine (transpersonal) (55). 3) We used the Reasons for Living Inventory for Adolescents (RFL-A), a 32-item self-report inventory (56). 4) We used the Life Events Questionnaire for Adolescents, a 39-item self-questionnaire developed by Newcomb et al. (57). 5) We used the Relationship Scales Questionnaire (RSQ) (58) to assess attachment styles.

All the questionnaires that were not available in Brazilian Portuguese (the Adolescent Coping Scale (ACS), the Spirituality Scale, the Reasons for Living Inventory, the Life Events Questionnaire for Adolescents, the Eysenck Questionnaire, the Ab-DIB, the Relationship Style Questionnaire (RSQ-ADO) and the DEP-ADO Detection of alcohol and drug problems in adolescents) were back translated by two Brazilian psychiatrists who were fluent in both languages and reviewed by a French native speaker who was also fluent in Portuguese.

### Ethics

Ethics approval was obtained for this study from CHU Charles Nicolle, France (2010 A00 330—39) and from the Group Ethics and Medical Research Committee of Sao Paulo University, Brazil (706939). Each participant gave their written informed consent prior to participation.

### Statistical Analysis

Statistical analyses were performed using R 3.4.0, and p-values less than 0.05 were considered significant. The goal of the analyses was to determine the relationship between geographic origin (Brazilian vs. French) and all sociodemographic and psychopathological variables. We started with univariate analyses to compare the two groups. All quantitative variables were described using the mean and standard deviation or frequencies and percentages. We used Student t-tests or Wilcoxon rank sum tests to compare normally distributed and nonnormally distributed quantitative variables, respectively, and we used Chi-squared or Fisher’s exact tests to compare normally distributed and nonnormally distributed qualitative variables, respectively.

Then, we aimed to compare French and Brazilian samples using multivariate analyses to determine the most robust variables. We selected the variables of interest based on the univariate analyses. We used a rather inclusive threshold (p < 0.2) to allow exploration of subtle effects (see **Table 2** below). The 12 variables that were included in the multivariate analyses were secure attachment style total score; Beck depression total score; reasons for living inventory total score; dependence total score; coping-social action; seeking spiritual support; self-discovery; lifetime suicide attempts; life events (autonomy); borderline disorder; anxiety disorders. Multivariate models are usually performed to assess a causal relationship. Here, the predictor variable to be explored was the geographic origin. However, we did not test the hypothesis that living in Brazil (or France) is a causal factor in being severely depressed or having attempted suicide several times, as this is not a plausible hypothesis.

Therefore, we explored each relevant clinical variable using a multivariate model as follows:

var1∼country+var2+…+var12

By doing so, we were able to calculate the effect of the geographic origin on a given clinical variable while taking into account the effects of other clinical variables. For example, we used the following formula for secure attachment style total score:

RSQ secure∼country+depression+reasons for living+DEP-ADO+coping social action+seeking spiritual support+self-discovery+lifetime suicide attempt+life events-autonomy+gender+borderline+anxiety (0/1)

The following formula was used for depression:

depression∼country +RSQ Secure+reasons for living+DEP-ADO+coping social +seeking spiritual support+self-discovery+lifetime suicide attempt+life events-autonomy+gender+borderline+anxiety (0/1)

In total, we ran 12 multivariate models to examine how each selected variable was related to geographic origin and to the other variables linked to geographic origin based on our univariate comparison. We used either linear regression with bootstrap resampling (boot package, R = 10,000) or logistic regression, depending on the nature of the explained variable. Missing values were imputed using a nonparametric random forest method (missForest package). Input variables were model variables, and some auxiliary variables were chosen for their correlation with model variables. In the *Results* section, we only present the effect of geographic origin on the selected variable (estimate, 95% confidence intervals; standard error, and p) for each model.

## Results

The average age among Brazilian adolescents and French adolescents was 14.42 (SD 1.7) years old and 14.73 (SD 1.29) years old, respectively. There were more boys in the Brazilian sample (71.1% girls vs. 28.9% boys), while there were more girls in the French sample (81.1% girls vs. 16.9% boys). Regarding differences in parents’ educational levels between the French and the Brazilians, there was a significant difference, with French parents reporting higher educational levels than Brazilian parents (**Table 1**).

**Table 1:** Sociodemographic and clinical characteristics.

Variable	French		Brazilian		*Test*	*p-value*
	(N = 320)		(N = 45)			
	*n*	%	*n*	%	**	**
**Gender**					χ² = 3.76	0.053
Boys	55	17.2	13	29		
Girls	265	82.8	32	71		
**Living arrangement**					χ² = 0.43	NS
Without both parents	187	58	21	47		
With both parents	133	42	24	53		
Mother’s level of education * Mean, SD	2	1 4	3	2 4	W = 2730	0.025
Father’s level of education * Mean, SD	2	2 2	3	2 4	W = 1729.5	0.028
**Repeated grade at school, Yes**	101	31.5	9	20	χ² = 2.7	NS
**Axis I diagnoses (DSM-IV-R)**						
Major depressive disorders	131	42.8	15	33	χ² = 1.45	NS
Adjustment disorder with depressed mood	238	77.8	41	91	χ² = 4.28	0.039
Anxiety disorders	88	28.0	22	49	χ² = 8.95	0.003
Disruptive and oppositional behavior	66	21.0	15	33	χ² = 3.06	NS
Eating disorders		2.3		11.1	F	0.011
**Method of suicide attempt**					F	0.005
Intoxication	242	75.6	27	60		
Laceration	20	6.2	11	24		
Strangulation	28	8.7	3	7		
Precipitation	13	4.0	1	2		
Others	17	5.5	3	7		
**Number of suicide attempts**					W = 5257.5	0.004
1	208	65	21	49		
2	59	18.4	5	12		
3	26	8.1	3	7		
>3	27	8.5	14	33		

* 1: primary and lower secondary school, 2: higher secondary school, 3: secondary professional school, 4: tertiary education (approximately 2-3 years more after high school diploma), 5: tertiary education (approximately 5-6 years more after high school diploma).

### Univariate Analyses

Brazilian adolescents presented more present adjustment disorders with depressed mood (41% vs. 33%, p=0.039), present anxiety disorders (48.9% vs. 27%; p = 0.003), and present eating disorders (11.1% vs. 2.3%; p = 0.011). No differences were found for present major depressive disorders and present disruptive and oppositional behavior (**Table 1**). Brazilian adolescents had significantly higher scores of BDI-II depressive symptoms (p < 0.001), BHS hopelessness (p = 0.038), and borderline psychopathology (93.2% vs. 71.6%; p = 0.002) compared to French adolescents (**Table 2**).

**Table 2:** Dimensional characteristics.

Variables	French		Brazilian		*Test*	*p-value*
	(N=320)		(N = 45)			
	*Mean*	*SD*	*Mean*	*SD*	**	**
**Reasons for Living Inventory for Adolescents**						
Family alliance	4.26	1.24	3.64	1.54	W=4660	NS
Suicide-related concerns	3.19	1.44	2.58	1.32	W=4511.5	0.008
Self-acceptance	3.78	1.27	3.49	1.50	W=5155	NS
Peer acceptance and support	4.34	1.24	3.23	1.48	W=3357	<0.001
Future optimism	4.07	1.22	3.86	1.44	W=5540	NS
Total score	4.09	1.18	3.28	2.06	W=4337	0.003
**Spirituality Scale**						
Spiritual beliefs	17.84	8.09	21.69	9.91	W=7833	0.021
Self-discovery	18.97	4.78	15.60	5.88	W=4212	<0.001
Self-awareness and collective consciousness	14.52	5.03	16.80	5.40	W=8002	0.01
Respect of others and environment	18.61	3.87	18.73	4.07	W=6787	NS
Total	69.97	16.63	76.16	20.03	W=7467	0.092
**Adolescent Coping Scale**						
Productive coping (total)	59.35	13.93	57.01	16.43	W=6158	NS
Focus on solving problem	51.12	16.06	58.04	18.89	t=2.34	0.023
Work hard and achieve	62.35	17.41	57.87	19.14	W=5845	NS
Focus on the positive	50.80	17.63	48.44	19.94	W=6110	NS
Seek relaxing diversions	73.45	19.66	70	20.30	W=6010	NS
Physical recreation	60.62	24.17	50.71	26.16	W=5260	0.013
Nonproductive coping (total)	54.42	12.10	56.29	9.70	W=7573	NS
Worry	52.66	17.42	57.69	20.64	W=7888	NS
Seek to belong	57.03	15.35	59.47	15.85	W=7199	NS
Wishful thinking	49.95	17.45	41.16	16.72	W=4686	0.002
Not coping	50.07	16.69	52.00	15.86	t=0.75	NS
Tension reduction	52.50	17.28	53.87	16.72	W=7168	NS
Ignore the problem	49.10	17.13	44.22	19.65	W=5670	NS
Self-blame	60.46	19.72	66.89	21.03	W=7967	NS
Keep to self	67.38	20.60	75.00	19.25	W=8336	0.016
Reference to others (total)	41.35	10.82	45.36	15.02	t=1.51	NS
Invest in close friends	58.51	17.61	49.96	17.57	W=4983	0.004
Seek social support	49.9	18.42	42.76	19.65	W=5228	0.011
Seek spiritual support	30.33	17.71	46.44	26.68	W=9228	<0.001
Social action	30.92	11.23	43.00	16.42	W=9646	<0.001
Seek professional help	39.66	18.99	44.67	21.01	W=7510	NS
**Life Events Questionnaire (total score)**	3.63	2.4	3.78	2.10	W=6917.5	NS
**Borderline psychopathology (Ad-DIB)**	19	14-26	28	19-37	W = 10158.5	<0.001
**Impulsivity (total score)**	12.18	4.68	13.57	3.69	W=7648	NS
**Dependence—DEP-ADO (total score)**	7.26	7.1	25.00	24.45	W=3363	0.001
**Depression (BDI total score)**	23	21.5	39	19.25	W = 9723	<0.001
**Hopelessness (BHS total score)**	9	8	12	3	W = 8098	0.038

DEP-ADO, Dependence Questionnaire for Adolescents; BDI, Beck Depression Inventory, BHS, Beck Hopelessness Scale; Ad-DIB, Abbreviated Self-Questionnaire of the Diagnostic Interview for Borderline Personality Disorder.

Regarding suicidal behaviors (C-SSRS), more Brazilian adolescents had a history of SA than their French counterparts [3.07 (SD 2.91) vs. 1.6 (SD 1.12), W = 861, p = 0.001]. Brazilian adolescents also presented more preceding behaviors, such as actively seeking the means to commit suicide or writing a letter to explain their suicide (χ² = 29.68, p < 0.001). The French sample had more definitive intentions than the Brazilian sample (χ² = 7.16, p = 0.007), while the Brazilian adolescents were characterized by more cases of deliberate self-injury (80% vs. 44%, χ² = 20.12, p < 0.001).

Both Brazilian and French adolescents had a detached relationship style (as measured by the RSQ), but Brazilians were significantly more detached (W = 8034.5, p < 0.001), less secure (W = 1899, p < 0.001), and more worried (W = 7762, p = 0.001) than French adolescents.

Regarding drug misuse (as measured by the DEP-ADO), Brazilian adolescents had significantly higher scores for alcohol and cannabis use (W = 3464, p < 0.001), whereas French adolescents scored higher on the use of other drugs (W = 2183.5, p = 0.02). Additionally, Brazilian drug users reported more consequences and overall more punctuation (W = 5423, p = 0.007).

Regarding life events, we assessed several domains: family, illness, sexuality, autonomy, deviance, and relocation. Brazilian adolescents reported significantly more autonomous life events (W = 811, p <0.001). Autonomous experiences, such as gaining a new group of friends or starting to make your own money, are usually perceived as positive and tend to increase with age in adolescence. We found no difference in the total score of life events between the two samples (W = 6917.5, p = 0.348).

Regarding coping skills (as measured by the ACS), Brazilian adolescents made greater use of “focusing on solving problems” (p = 0.023), “social action” (p < 0.001), “keeping to self” (p = 0.016) and “seeking spiritual support” (p < 0.001) strategies, while French adolescents scored significantly higher on “social support” (p = 0.011), “investing in close friends” (p = 0.004), “wishful thinking” (p = 0.002), and “physical recreation” (p = 0.013) items (**Table 2**).

French adolescents scored higher on the reasons for living inventory. Regarding the subcategories, French adolescents scored higher on the “peer acceptance and support” (p < 0.001), family alliance (p = 0.017), and suicide-related items (p = 0.008). We found no difference between the two groups on the “self-acceptance” and “future optimism” items (**Table 2**).

Regarding the spirituality scale, French adolescents scored higher on “self-discovery” (p < 0.001), while the Brazilians reported higher scores in “spirituality and beliefs” (p = 0.021) and “respect of others and environment” (p = 0.01). We found no difference between the two groups for “self-awareness and collective consciousness” (**Table 2**).

### Multivariate Models

Based on the results from univariate analyses, we developed 12 multivariate models to examine each selected variable’s relationship to French or Brazilian origin and to the other selected variables linked to geographic origin (see *Methods* section). The list of the variables and the results of the multivariate models are summarized in **Table 3**. Four variables were significantly explained by membership in the Brazilian sample, namely, “seeking spiritual support” (ACS) (β = -16.74, [95% CI -28.77 - -5.15], SE = 6.13, p <0.001), “social action” (ACS) (β = -13.98, [95% CI -20.61 - -6.85], SE = 3.47, p <0.001) and “dependence” (DEP-ADO total score) (β = -15.98, [95% CI -22.58 - -11.23], SE = 2.97, p <0.001), and life events (autonomy) (β = -0.27, [95% CI -0.51 - -0.03], SE = 0.12, p 0.032). Two variables were significantly explained by being part of the French sample, namely, “secure attachment style” (RSQ) (β = 12.68, [95% CI 11.37 - 14.33], SE = 0.78, p < 0.001) and a higher score in the Beck Depression Inventory (BDI II) (β = 9.16, [95% CI 3 - 15.46], SE = 3.22, p <0.001) (**Table 3**).

**Table 3:** Multivariate models to explain each selected variable by the French or Brazilian origin.

	Estimate	95% CI	SE	p-value
Secure attachment style (RSQ)	12.68	[11.37; 14.33]	0.78	<0.001
Beck Depression (BDI II total score)	9.16	[3; 15.46]	3.22	<0.001
Reasons for Living Inventory (total)	-0.03	[-0.49; 0.56]	0.27	NS
Dependence (DEP-ADO total score)	-15.98	[-22.58; -11.23]	2.97	<0.001
Social action (ACS)	-13.98	[-20.61; -6.85]	3.47	<0.001
Seek spiritual support (ACS)	-16.74	[-28.77; -5.15]	6.13	<0.001
Self-discovery (Spirituality Scale)	1.57	[-0.29; 3.71]	1.02	NS
Lifetime suicide attempts	-0.11	[-1.05; 0.89]	0.48	NS
Life Events (autonomy)	-0.27	[-0.51; -0.03]	0.12	0.032
Gender (F/M)	-1.11	[0.07; 1.49]	0.77	NS
Borderline Psychopathology (No/Yes)	1.01	[0.21; 33.09]	1.28	NS
Anxiety Disorders (No/Yes)	-0.49	[0.17; 2.27]	0.66	NS

ACS, Adolescent Coping Scale; RSQ, Relationship Scale Questionnaire; DEP-ADO, Dependence Questionnaire for Adolescents.

## Discussion

In this study, we compared two clinical samples of suicidal adolescents to determine the main risk and protective factors associated with SA. To our knowledge, this is the first comparative study on suicidal behavior among adolescents between a European and a Latin American country. We first hypothesized that adolescents in the two groups would not present any difference in the main risk factors associated with suicidal behavior in adolescence. Indeed, the two samples presented very similar characteristics regarding major depressive disorders, disruptive and oppositional disorders, impulsivity, borderline traits, and use of substances, although the Brazilian sample had higher rates of psychopathology in several of these diagnoses (anxiety, borderline traits, use of substances, and suicide behavior). The high rates of psychiatric diagnosis found in the study were consistent with the well-established literature that has shown an association between the occurrence of suicidal behavior in adolescents and psychiatric diagnoses in more than 90% of cases (13). Borderline psychopathology was found in an extremely high percentage of both Brazilian and French adolescents. Studies have demonstrated that this diagnosis is the greatest source of suicidal behaviors in adolescents (59, 60).

The higher psychopathology scores of the Brazilian adolescents in almost all the clinical disorders constituted the major clinical difference between the two samples. A study conducted in Sao Paulo (Megacity Mental Health Survey) revealed that mental disorders are notably prevalent in this city, with an estimated prevalence of 10% of severe cases, which is the largest proportion of severely affected subjects between the countries that also took part in the initiative run by the WHO (61–64). Major depression emerged as one of the most prevalent disorders, with a higher estimated prevalence than has been seen elsewhere in other participating countries (65), which offers evidence on the burden of mental health in a developing country (66). Although the megacity study was conducted only on adults, there are also prevalence studies in children and adolescents, with results showing slightly but significantly higher levels of psychopathology in Brazil than in other countries (34, 35).

Regarding dimensional characteristics, such as attachment relationships, spirituality and coping strategies, we hypothesized that these variables would be influenced by geographical origin. Our second hypothesis was also confirmed. Indeed, there were significant differences in scores for spirituality, reasons for living, life events, attachment style and coping skills. Four protective factors remained significant in the multivariate models: attachment style, spiritual coping, and coping with social action and life events. Having a “secure style of attachment” was a protective factor associated with membership in the French cohort. An increased use of the spiritual type of coping and coping through social action was a protective factor associated with membership in the Brazilian cohort. These results are complex. We believe that *per se*, the two protective factors have specific effects. The protective effect of secure attachment in adolescent psychopathology in general [e.g., (67)] and regarding suicidality specifically (68) has been shown in several studies. Additionally, it is known from the literature that the relationship between religiosity and suicidal behavior is complex and often protective, although it can vary among different cultures and populations (69). Coping through social action and spiritual coping are both categorized in factor 3 of the ACS, which groups the coping styles characterized by reference to others, whether peers, professionals or deities (54, 70). The “use of others” as a resource that is compatible with a lack of any other resources (social and professional support or recreative activities) and with a more insecure attachment style is found in the Brazilian sample. Although less significant, “autonomous” life events were more common in the Brazilian sample. Some negative life events are related to psychological symptoms; however, personal “autonomous” life events were not associated with dysfunction (57, 71).

It is also important to note that empirical data strongly suggest the association of insecure attachment and substance abuse (72), since secure attachment is a well-described protective factor for drug use (73). These findings are likely related to the differences found in the Brazilian and French samples regarding substance use and attachment. Similarly, there are also some established relationships between religion and style of attachment, in which religiosity serves as a compensatory strategy for insecure attachment (74, 75). In a cross-sectional study using an adolescent sample (76), the results suggested that religiosity played the role of offering emotional support for insecure attachment in childhood. This association was partially supported by a similar study conducted more recently in 2016 (77): the authors found similar results in the cross-sectional arm but found mixed results after the 18-month follow-up period. Interestingly, insecure attachment has also been linked to instability in religiousness, particularly to sudden distress-related religious conversions (78). Brazil has been experiencing profound changes in regards to religiosity in recent decades, with a high number of new conversions to evangelical churches and a decline in absolute numbers of Catholics. The proportion of evangelical individuals in Brazil increased from 10.8% in 1991 to 21% in 2000 and to 34.3% in 2010 (79). The presence of insecure attachment and spiritual coping styles found in the Brazilian sample and confirmed by the multivariate analyses may suggest that religiosity in the Brazilian sample may serve as an emotional compensation strategy. The associations between recent changes in Brazilian society and the occurrence of insecure attachment need to be further investigated. The causes and consequences of these phenomena are complex, but we suggest that they may contribute to our hypotheses of emotional compensation in the Brazilian sample of adolescents with a history of SA.

## Limitations

First, the Brazilian sample was much smaller than the French sample. The sample in Brazil came from an emergency department of a public university hospital, which suggests only patients with the most severe cases were included. In addition, only one recruitment site was involved in Brazil, while there were five sites in the French sample. This limits the generalization of the findings to all Brazilian adolescents. Such limitations are related to the difficulties in collecting psychiatric outcome data in a middle-income country (80). However, the Brazilian centre was not chosen randomly (see *Method* section). Second, modalities to run the interviews were not similar. French adolescents were assessed during an inpatient stay. Brazilian patients were asked to participate when they were in the emergency department and then contacted later by the research team and interviewed within a month.

Third, regarding substance abuse disorders, we only obtained the results from a questionnaire (DEP-ADO). The DEP-ADO is a questionnaire that assesses alcohol and drug use among adolescents and does an initial screening for problematic or at-risk consumption. It can be administered in face-to-face or self-report modes both for screening and for research and epidemiological monitoring purposes. Finally, while all questionnaires used herein have been validated in French, some Brazilian/Portuguese questionnaires were only translated/back translated for the purpose of this study, and the psychometric properties of these translated versions have not been examined.

## Conclusions

Although French and Brazilian adolescents present similar rates of disorders, the Brazilian sample presented higher levels of psychopathology (except for depression severity). In addition, Brazilian adolescents had a more insecure pattern of attachment and used religious coping more often than their French counterparts. These differences should be further studied in the future. There are previous studies suggesting that religion may compensate for the social vulnerabilities found in a middle-income country (40). More transcultural studies may help elucidate these findings.

## Data Availability Statement

Datasets are available upon request to the corresponding author, BM (bojan.mirkovic@chu-rouen.fr).

## Ethics Statement

The studies involving human participants were reviewed and approved by Ethics approval was obtained for this study from CHU Charles Nicolle, France (2010 A00 330 - 39) and from the Group Ethics and Medical Research Committee of Sao Paulo University, Brazil (706939). Written informed consent to participate in this study was provided by the participants’ legal guardian/next of kin.

## Author Contributions

NR and BM were responsible for data collection, preparing the first draft of the manuscript and finishing the final version of it, including the coauthors’ comments and suggestions. JS and AC were involved in the collection and analysis of data. HP was responsible for the data analyses. TF, DS, PG, and DC were responsible for the supervision of data collection and analyses and thoroughly revised all the versions of the manuscript.

## Conflict of Interest

The authors declare that the research was conducted in the absence of any commercial or financial relationships that could be construed as a potential conflict of interest.
